# Preparing to launch the ‘Thinking Healthy Programme’ perinatal depression intervention in Urban Lima, Peru: experiences from the field

**DOI:** 10.1017/gmh.2018.32

**Published:** 2018-12-18

**Authors:** B. S. Eappen, M. Aguilar, K. Ramos, C. Contreras, M. C. Prom, P. Scorza, B. Gelaye, M. Rondon, G. Raviola, J. T. Galea

**Affiliations:** 1 Socios En Salud, Lima, Peru; 2 Department of Psychiatry, Massachusetts General Hospital, Boston, USA; 3 Department of Psychiatry, Columbia University, New York, USA; 4 Department of Epidemiology, Harvard T. H. Chan School of Public Health, Boston, USA; 5 Department of Medicine, Cayetano Heredia Peruvian University, Lima, Peru; 6 Partners In Health, Boston, USA; 7 Department of Global Health and Social Medicine, Harvard Medical School, Boston, USA; 8 School of Social Work, College of Behavioral and Community Sciences, University of South Florida, Tampa, USA

**Keywords:** Intervention, interventions, low-intensity, perinatal depression, Peru, WHO

## Abstract

**Background.:**

An estimated 19–25% of perinatal women in low- and middle-income countries are affected by depression which, untreated, is associated with multiple health problems for mothers and children. Nonetheless, few perinatal women have access to depression care. The Thinking Healthy Programme (THP), promoted by the World Health Organization (WHO), is an evidence-based, non-specialist delivered depression intervention that addresses this care gap. However, the WHO THP manual explains intervention delivery but not the antecedents to implementation. Here, we describe a principled, planned approach leading to the implementation of THP in Lima, Peru by the non-profit organization Socios En Salud with community health workers (CHW) to inform its implementation in other settings.

**Methods.:**

The Replicating Effective Programs (REP) framework guided THP implementation, following four phases: (I) pre-conditions; (II) pre-implementation; (III) implementation; and (IV) maintenance and evolution. This paper centers on REP phases I and II, including (1) documented high perinatal depression rates in Peru; (2) designation of perinatal depression as a government priority; (3) THP Implementation Team orientation and training; (4) data collection plan development; (5) public health system coordination; (6) CHW selection and training; and (7) THP launch.

**Results.:**

Between December 2016 and March 2017, a THP training program was developed and seven CHW were trained to deliver the intervention to 10 perinatal women, the first of whom was enrolled on 17 April 2017.

**Conclusions.:**

THP was rapidly implemented by a community-based organization with no prior experience in delivering non-specialist perinatal depression care. The steps followed may inform the implementation of THP in other settings.

## Background

Nearly 322 million people live with depression – 48 million in the Americas alone – a number which has increased yearly since 1990 (GBD, 2013 DALYs & HALE Collaborators, 2015). Now the third leading cause of years lived with disability worldwide (GBD, 2015 Disease and Injury Incidence and Prevalence Collaborators, 2016), depression affects not only psychological wellbeing, but is linked to increased risk for suicide, dementia, and premature mortality from co-existing physical disorders (Reynolds & Patel, 2017).

Women globally are disproportionally affected by depression compared with men at a rate of nearly 2:1 (Kessler, 2003). For perinatal women, depression rates are estimated at 7–15% in high-income countries (Evans *et al*. 2001; Grote *et al*. 2010) and 19–25% in low- and middle-income countries (LMICs) (Rahman *et al*. 2003). Antenatal depression alone increases the risk of negative health-related behaviors and obstetric complications including poor nutrition, increased substance use, inadequate prenatal care, pre-eclampsia, low birth weight, preterm delivery, postpartum depression, and suicide (Gelaye *et al*. 2016). Moreover, a mother's untreated depression can lead to impairments of the child's emotional, social, and cognitive development; rarely infanticide may even occur (Vigod & Stewart, 2017).

Peru is an upper middle-income country with a perinatal depression prevalence rate estimated between 24% and 40% (Aramburú *et al*. 2008; Luna Matos *et al*. 2009); however, only approximately 10% of all women requiring mental health services access care (MINSA, 2016). Peru's Ministry of Health guidelines specify that pregnant women have at least one consultation with a psychologist (MINSA, 2011), but the application of this policy is unknown. In 2015, the Peruvian Ministry of Health began expanding mental health services delivery from a predominantly tertiary-level system to the primary care level (Miranda *et al*. 2017) guided by the World Health Organization's (WHO) Mental Health Gap Action Programme (mhGAP) (WHO, 2008). The mhGAP provides evidence-based guidelines and tools to assist LMICs with the expansion of mental health services to primary care settings. In addition, the Ministry of Health initiated multiple strategic alliances with non-governmental organizations to assist with patient identification, education, referral, and delivery of low-intensity, non-pharmacological interventions for common mental illnesses, such as depression (Toyama *et al*. 2017).

It was in this context that the Peruvian branch of the international organization Partners In Health, known locally as ‘Socios En Salud’ (SES), choose to implement the WHO's ‘Thinking Healthy Programme’ (THP), a low-intensity, non-pharmacological intervention for psychological management of perinatal depression (WHO, 2015). The THP is one of three evidence-based interventions for the treatment of depression included with the mhGAP. These interventions are ‘low intensity’ because they are designed to use fewer resources than more traditional psychological interventions and can be effectively delivered by people without previous mental health training. THP is a cognitive behavioral intervention that can be delivered to perinatal women in their homes by trained community health workers (CHW), beginning during the last trimester of pregnancy and lasting until approximately 10 months postnatal. Several countries have evaluated or implemented THP, including Pakistan (Rahman, 2007; Rahman *et al*. 2008), India (Sikander *et al*. 2015), Vietnam (Fisher *et al*. 2014), as well as Bangladesh, Nigeria, and Bolivia (Mental Health Innovation Network, 2017); however, we are unaware of published reports describing the antecedents to implementing THP outside of a clinical trial setting.

The full THP intervention consists of 16, 1-h sessions grouped into five modules: (1) preparing for the baby (sessions 1–4); (2) the baby's arrival (sessions 5–7); (3) early infancy (sessions 8–10); (4) middle infancy (sessions 11–13); and (5) late infancy (sessions 14–16). The WHO's THP delivery manual describes the intervention in detail and provides step-by-step guidelines for delivering each of the sessions. However, there are a series of activities prior to intervention implementation for which no published guidance yet exists. For example, though THP was designed to be delivered by people with no prior mental health training, an understanding of perinatal depression and having basic counseling skills are prerequisites to its successful implementation. Organizations may wonder where to begin if they, like SES, lack prior experience in the delivery of non-specialist mental health interventions. Our aim is to describe a principled, planned approach developed by SES to implement THP to inform implementation in other settings. In this paper, our focus is limited to the antecedents leading up to the launch of THP in Lima, Peru.

## Methods

**Fig. 1.**
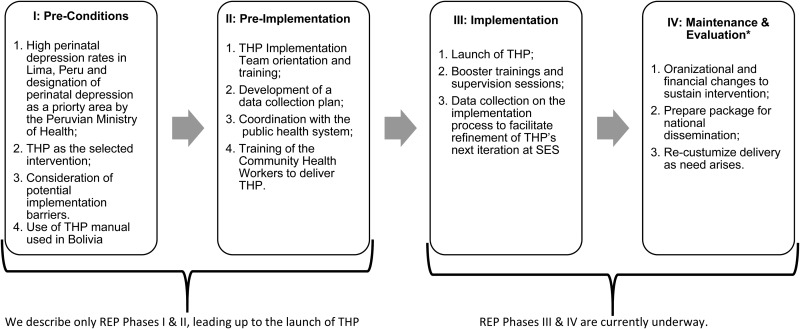
A complete Replicating Effective Programs (REP) framework for the implementation of the Thinking Healthy Programme in Lima, Peru. *Steps for REP Phase IV are taken, as written, from Kilbourne, 2003.

We used the Replicating Effective Programs (REP) framework (see Kilbourne *et al*. 2007) to guide our THP implementation plan. The complete REP framework consists of four phases: (I) pre-conditions; (II) pre-implementation; (III) implementation; and (IV) maintenance and evaluation (Fig. 1). In the remainder of this article, we describe a process undertaken at SES between December 2016 and March 2017 leading to the implementation launch of THP in Lima, Peru corresponding to REP phases I and II. The implementation of THP (REF phase III) is briefly addressed but only from the planning perspective rather than the results of the actual implementation. Both REP phases III and IV will eventually draw from the data collection and supervisory structures described below, but because the intervention is currently in the initial implementation phase, we do not address these aspects in detail here.

### REP phase I: preconditions

Kilbourne *et al*. (2007) describe the pre-conditions phase as: (1) the identification of the need for an intervention to address a given problem or issue; (2) the selection of an intervention that both addresses the identified problem and fits the local setting; (3) identifying implementation barriers; and (4) the production of an intervention manual.

1. Identification of the intervention need

Need for a perinatal depression intervention included a high prevalence of perinatal depression in Lima, Peru (Aramburú *et al*. 2008; Luna Matos *et al*. 2009) and the designation of perinatal depression as a priority area with dedicated funding by the Peruvian Ministry of Health (Toyama *et al*. 2017). Further, in August of 2014, SES met with leadership at the Peruvian National Institute of Mental Health where an inter-institutional agreement was signed to promote collaboration between the organizations to increase access of community-based mental health services in Peru (Instituto Nacional de Salud Mental, 2014). In that meeting, SES’s commitment was to develop mental health services delivered outside of medical centers by non-specialized persons like CHW.

1. Selection of the intervention to address the issue

THP was selected because it was an evidence-based perinatal depression intervention promoted by the WHO and a natural fit for SES as an organization and the community it serves. THP is designed to be delivered by CHW, and since its inception, SES has worked closely with CHW, first in the delivery of home-based multidrug-resistant tuberculosis treatment (Mitnick, 2003), and later in HIV treatment (Muñoz, 2010) and most recently in early childhood development (Nelson, 2018). In each case, SES and CHW articulated care to strengthen and support the Ministry of Health rather than to replace or duplicate pre-existing services. Consequently, SES and the community were already primed for THP because the approach of potentiating non-specialists to deliver a health intervention is part of SES’s ‘DNA’ and the community was accustomed to receiving CHW-delivered health interventions. Indeed, though not formally part of the Peruvian public health system, CHW have an undeniable role in extending the reach of health care beyond established health centers, especially by promoting healthy practices in areas including maternal health, nutrition, anemia, and hygiene (MINSA, 2015).

1. Consideration of potential implementation barriers

Regarding potential barriers to THP, the biggest potential barrier could be time intensity and duration of the intervention (16 sessions over 10 months) but we did not conduct any formal assessments on this or other potential barriers. Instead, our plan was to begin small, with only one health center and 10 perinatal women so that implementation barriers specific to our setting could be discovered and addressed in practice without having to add the extra time and expense of a formative evaluation phase. Our plan was in harmony with the mhGAP's stated approach of disseminating interventions that are both evidence-based and considered feasible in low-resource settings (WHO, 2008).

1. Production of an intervention manual

The final pre-condition – the production of an intervention manual – was unnecessary because we learned that the non-profit organization CBM (formerly Catholic Blind Mission) had implemented THP in Bolivia (Mental Health Innovation Network, 2017) and had a Spanish language, culturally adapted version of the THP manual that they agreed to share with SES. Because Peru and Bolivia are neighboring, Andean countries, there are a number of cultural similarities (e.g. Spanish language, many foods, clothing, religious beliefs) that made the Bolivian THP manual readily understable in Peru. The Bolivian manual, apart from being in Spanish, had improved cultural specificity over the generic WHO version by using examples and graphic illustrations relevant to South America (e.g. replacing drawings of women in Pakistan found in the original WHO manual with drawings of women in typical Andean dress common in Bolivia); however, all core intervention content was unchanged from the original WHO manual.

### REP phase II: pre-implementation

Following the pre-condition phase, we planned the implementation of THP in accordance with REP phase II which consisted of four steps: (1) THP Implementation Team (IT) orientation and training and external expert consultation; (2) development of a data collection plan; (3) coordination with the public health system; and (4) training of the CHW to deliver THP.

1. IT training

An IT was formed consisting of SES staff and affiliate members to guide the training process (see Table 1 which summarizes the key activities, IT members and timeframe of the SES THP implementation). We began by ensuring a clear, common understanding of THP among the IT led by the coordinators of SES’s mental health program and maternal–child health program. The IT met weekly during December 2016 to read the entire manual aloud and discuss the intervention content and potential implementation feasibility issues. All concerns were noted for external consultation via email and teleconference with the THP developers in Pakistan [part of the Human Development Research Foundation (HDRF), a non-profit research, education, and service organization] and the CBM consultant (responsible for guiding THP implementation in Bolivia).

**Table 1.** Key activities, actors and timeframe for the implementation of the Thinking Healthy Programme in Lima, Peru

Activity	Actors (author initials where applicable)	Timeframe
Initial consultations and selection of intervention	SES Deputy Director (JG) Partners In Health Mental Health team/Academic mentor for Abundance Fellowship (GR) SES Interventions Director (CC) Human Development and Research Center, Pakistan	September 2015–July 2016
Collaborative training and preparation	Thinking Healthy Implementation Team (IT): Socios En Salud (SES) Mental Health Program Coordinator (MA) SES Maternal-Child Health Program Coordinator (KR) SES Interventions Director (CC) SES Deputy Director (JG) SES Mental Health team intern (BE)* Partners In Health Mental Health team (GR)* Massachusetts General Hospital psychiatry resident (MP)*	December 2016–March 2017
Data collection consultations	Harvard School of Public Health (BG) Universidad Peruana Cayetano Heredia (MR) Colombia University (PS)	January 2017
Data collection planning and instrument adaptation	Thinking Healthy Implementation Team (see above)	January 2017–April 2017
Implementation consultations	Human Development and Research Center, Pakistan CBM (formerly Catholic Blind Mission)	February 2017
Adaptation of training materials	Thinking Healthy Implementation Team	February 2017–March 2017
Coordination with health system	Director of mental health, Ministry of Health Medical director of Ministry of Health administered health center	January 2017
Training	Thinking Healthy Implementation Team Community Health Workers	March 2017
Enrollment	Maternal-Child Health Program Coordinator (KR) Mental Health Program Coordinator (MA)	April 2017–August 2017
Implementation	Community Health Workers	April 2017–(ongoing)
Supervision	Mental Health Program Coordinator (MA) Community health workers Partners In Health Mental Health team (GR)	April 2017–(ongoing)

*BE, GR, and MP did not participate in the group reading of the THP manual.

The IT was unsure how to structure the initial CHW training (e.g. overall approach, training duration, use of roleplays, and supplementary didactic materials). A related concern was how to prepare for specific scenarios that could arise during the THP implementation (e.g. CHW difficulty identifying negative thoughts; handling missed or abbreviated sessions); and other key considerations (e.g. determining inclusion criteria for mothers; family member involvement; managing local/Peruvian-specific beliefs regarding motherhood and child rearing in relation to THP; providing THP in conjunction with pharmacological interventions for severe depression; and use of THP monitoring and evaluation tools).

Each of these concerns was discussed in early 2017 with THP implementers at HDRF and CBM. A major advantage to talking with these experienced organizations was the ability to receive not only previously used materials but also the benefit of talking to people with actual experience in THP implementation. HDRF shared with the IT the THP training materials used in Pakistan, which included an electronic presentation of over 200 slides that guided a CHW training delivered over 5 days. The presentation included information on perinatal depression and its effect on women; an explanation suitable for laypersons of THP and its theoretical underpinnings; intervention delivery instructions; and roleplays to practice THP delivery. The IT decided to use the HDRF training presentation as received with only minor adaptations (apart from translation from Spanish) including the alteration of the training schedule and tailoring some of the roleplay exercises so that they would be understood in Peru (e.g. changing names used in Pakistan to Spanish names; changing example situations and drawings/illustrations from rural Pakistani to those relevant to Lima, Peru; see step 4, CHW training, below). Coupled with the THP manual provided by CMB (described in REP phase I, pre-conditions), SES did not need to design any new implementation materials representing savings in both cost and time to implementation launch.

Regarding the specific scenario questions, we learned that while THP is evidence-based and undergirded by a theoretical framework (cognitive–behavioral theory) that should be adhered to, it is also meant to be flexible in its delivery. Therefore, if a session required repeating because a mother did not fully grasp the concept taught, or if the session delivery schedule needed to be condensed or expanded, this was permissible so long as the core elements of the intervention were still being delivered. Likewise, while THP should be compatible with pharmacological interventions (e.g. antidepressants), consultation with the health center medical provider as part of the care team can determine whether THP should be delayed or suspended depending on the mother's specific mental health history.

Finally, questions regarding beliefs regarding motherhood and childrearing in Peru as they related to THP – namely, that it is a mother's sole responsibility to care for her baby while THP encourages the involvement of other family members as key supports for the health of both the mother and baby – were similarly framed in a flexible, non-confrontational way. With this cultural more, as with others, the overarching rule was to never confront, judge, or attempt to convince a mother or her family of a new approach that may be unwanted or create feelings of inadequacy as to what constitutes being a ‘good mother’. Instead, the recommendation was to gently suggest the participation of other family members (e.g. helping to feed, bathe, or soothe the infant) as something that other mothers have found useful. Our training therefore coached CHW to meet the mother ‘where she is at’, allowing her to decide what works best for her specific situation while at the same time supporting her in finding alternatives to negative thoughts, feelings, and actions.

1. Data collection planning

While THP does not include a data collection component, we sought to understand key aspects of the intervention implementation, as well as select characteristics of the initial cohort of the 10 mothers enrolled. Our rationale for planning data collection in this THP pilot was to prepare for scaling the intervention to the country level and providing data that could inform innovations to the intervention (e.g. the introduction of future mobile-health components to THP). Also, since we anticipated the need to modify the intervention delivery schedule in some cases (by adding, subtracting, or splitting some sessions), we wanted to track the extent to which such changes occurred. Emphasis was placed on data to characterize the implementation process (including time spent, costs incurred, number of sessions delivered, etc.) and the mothers who participated.

Understanding that ours is not a research study, we endeavored to be parsimonious with the quantity of data collected because we did not want to burden either the mothers, CHW, or SES staff. We therefore consulted with the HDRF team about intervention-specific data and with two perinatal health experts regarding the data collected and instruments used to arrive at the minimal amount data needed to inform future plans. Existing and/or standardized instruments validated in Peru were used whenever possible. Except for a session encounter form to track key aspects of the THP session delivered, all data were planned to be collected by SES staff to allow the CHWs to focus only on the implementation of the THP intervention.

Table 2 lists all forms planned for use, sorted by implementation process data and data specific to the mother participant, listing the corresponding THP manual module, form purpose, whether the form was pre-existing at SES or specific to THP, and when and by whom the form will be applied. Briefly, the implementation process data planned to be collected included: number and duration of completed sessions; reasons for missed sessions; staff/CHW transportation costs incurred; mothers’ completion of assigned tasks outside of sessions; type and quantity of problems encountered; requests for follow-up from the IT and health professionals; and CHW THP delivery performance.

**Table 2.** Data collection forms* planned for use during the implementation of the Thinking Healthy Programme in Lima, Peru

Form (abbreviation where applicable)	Corresponding THP manual module	Assesses, screens, and/or tracks	Form specific to mothers receiving THP (yes/no)	When applied	Applied by whom
Implementation process data					
Visit tracking	Completed after every session attempted/completed with the mother	Visit type, duration, deviations, costs, completion of THP exercises, mother's wellbeing, and referral needs	Yes	At each THP visit where intervention delivery is planned	Community health worker
Community health worker evaluation	1–5	Community health worker performance during THP session delivery	Yes	Applied at least once during each of the five THP modules	Staff psychologist observing the community health worker
Participant data					
Demographics	Applied prior to initiation of THP module 1, upon first contact with the perinatal mother	Socio-demographic characteristics; family composition; housing conditions	No	At first contact with the perinatal mother	Staff psychologist
Self-Reporting Questionnaire (SRQ-18)	Applied prior to initiation of THP module 1, upon first contact with the perinatal mother	Psychiatric disturbances	No	At first contact with the perinatal mother	Staff psychologist
Patient Health Questionnaire (PHQ-9)	Applied prior to initiation of THP module 1 and then after completion of modules 2, 3, 4, and 5	Depression	No	Week 30–33 of pregnancy and then at postnatal week 5 and postnatal months 4, 7, and 10	Staff psychologist
Edinburgh Postnatal Depression Scale (EPDS)	Applied prior to initiation of THP module 1 and then after completion of modules 2, 3, 4, and 5	Perinatal depression	Yes	Week 30–33 of pregnancy and then at postnatal week 5 and postnatal months 4, 7, and 10	Staff psychologist
Pittsburgh Sleep Quality Index (PSQI)	Applied prior to initiation of THP	Sleep quality	Yes	Week 30–33 of pregnancy	Staff psychologist
Adverse Childhood Experience Questionnaire (subset of full questionnaire) (ACE)	Applied prior to initiation of THP	Sexual and physical abuse of during childhood	Yes	Week 30–33 of pregnancy	Staff psychologist
Post-traumatic stress disorder Checklist-Civilian Version (PCL/C)	Applied prior to initiation of THP	Post-traumatic stress disorder	Yes	Week 30–33 of pregnancy	Staff psychologist
WHO Intimate Partner Violence Survey (subset of full questionnaire)	Applied prior to initiation of THP	Intimate partner violence	Yes	Week 30–33 of pregnancy	Staff psychologist
Physical activity	Applied prior to initiation of THP	Amount of physical activity in daily life	Yes	At first contact with the perinatal mother	Staff psychologist
Substance use	Applied prior to initiation of THP	History of psychoactive substance use	Yes	Week 30–33 of pregnancy	Staff psychologist
Baby medical history	Applied prior to module 2	Baby's health status	No	At birth, taken from medical records	Staff nurse
Baby follow-up	2–5	Baby's health center check ups	No	Week 30–33 of pregnancy and then at postnatal week 5 and postnatal months 4, 7, and 10	Community health worker
Mother's health status	1–5	Mother's physical health, including physical activity using select questions from the WHO Global Physical Activity Surveillance questionnaire	Yes	Week 30–33 of pregnancy and then at postnatal week 5 and postnatal months 4, 7, and 10	Staff psychologist
Health clinic follow-up	1–5	Mother's health center perinatal check ups	No	Week 30–33 of pregnancy and then at postnatal week 5 and postnatal months 4, 7, and 10	Community health worker

Data planned to be collected specific to mothers included (in-house forms used except where noted): socio-demographic characteristics; family composition; housing conditions; current psychiatric disturbances using the Self-Reporting Questionnaire (SRQ-18); depression symptom severity and changes using both the Patient Health Questionnaire (PHQ-9) and the Edinburgh Postnatal Depression Scale (EPDS); sleep quality using the Pittsburgh Sleep Quality Index (PSQI); history of sexual or physical abuse during childhood (section 10 of the WHO Domestic Violence Survey); symptoms of post-traumatic stress disorder (PTSD Checklist-Civilian Version); current or past history of intimate partner violence (using a subset of the WHO Intimate Partner Violence Survey); amount of physical activity (select questions from the WHO Global Physical Activity Survey); history of substance use; and data collected tracking visits to the health center for perinatal care and the baby's health.

Both the PHQ-9 and the EPDS were planned for depression screening and monitoring due to only a moderate agreement between the instruments to detect suicidal ideation among pregnant women as reported in a study conducted in Lima, Peru among 1517 pregnant women (Zhong *et al*. 2015). Further, while the PHQ-9 is validated in Peru (Calderón, 2012), it is not routinely used to screen for depression in perinatal women. However, SES routinely uses the PHQ-9 for depression screening among all adult populations and ideally would extend its use among perinatal women. By using both the EPDS and the PHQ-9, we could ensure the availability of data that could be shared with a mother's medical provider (i.e. the EPDS) while at the same time collect data using the PHQ-9 in this population.

1. Health system coordination

The implementation of THP in Peru was planned at the outset as an extension of the existing continuum of perinatal care delivered by the Ministry of Health. SES has more than 20 years of collaborative work with the municipality and health centers located in Carabayllo, located in the northern outskirts of Lima, Peru. Carabayllo [population 301978 in 2015 (INEI, 2018)], the first and largest of Lima's 43 districts, is home to many migrants from the interior of the country and is characterized by high levels of unemployment and poverty. Lack of potable water, sanitary sewage, and electricity is not uncommon, especially in more remote parts of the district. Access to health services is made difficult by Carabayllo's terrain, much of which consists of steep, rocky cliffs where thousands of families have constructed simple homes which are often accessible only by long, steep staircases or precarious dirt paths cut into the hillside. Descending the hills to arrive at nearby health centers is difficult, especially for the young, old, pregnant, and ill.

THP represented the first time that SES would work with CHW in the delivery of an in-home perinatal depression intervention, but the approach was the same: we began by coordinating with the local health center. Staff from SES’s maternal–child health team met with the medical director of one of the health centers in which maternal–child health work was already being conducted to offer THP as an additional service. With the health center's Director's approval, a list was created of all pregnant women receiving care at the clinic and participating in SES’s maternal child health program. Women on the list were ordered by expected delivery date. Because THP begins during the last trimester of a woman's pregnancy, the first 10 women expected to enter their last trimester were invited to participate. There were no other exclusion criteria. All 10 women invited agreed to participate in our THP pilot as they entered their third trimester of pregnancy.

1. CHW training

THP assumes that the CHW delivering the intervention have a basic understanding of perinatal and infant health and knowledge of the public health and community resources available to assist women. At the health center where the THP pilot was planned, seven CHW (all women) were collaborating with SES in the maternal–child health program and all were invited to participate in THP. These CHW had been trained by SES in maternal–child health (e.g. prenatal health, the birthing process, breastfeeding, infant care, etc.) and were providing accompaniment to the pregnant mothers who were also invited to participate in THP. Since THP is expressly designed for deployment by non-specialist workers, the only criterion for a CHW's participation in the THP training was an interest in learning to deliver a depression intervention to perinatal women.

The THP training from HDRF was structured over 5 days, but the IT initially decided on a denser schedule structured over 3 days (9:00–14:00 h) to minimize the impact on the CHWs’ work and personal schedules. Nonetheless, we found after the first day that ending at 14:00 h still conflicted with other duties that the CHWs had; therefore, we restructured the training over four shorter days to better accommodate schedules. The training content provided by the HDRF and consisted of the following core elements: Day 1: introductions and ice-breaker; description of perinatal depression; stigma toward perinatal depression; negative thoughts held by perinatal mothers; THP's central principles; qualities of good CHWs; and an overview of the first THP session. Day 2: the mother's wellbeing; the three THP steps (identifying negative thoughts, replacing them with alternative thoughts, and practicing healthy thoughts and behaviors); and the THP health calendar used for mood, diet, sleep, and behavioral activation tracking. Day 3: the mother–infant relationship; the relationship between the mother and the people around her; and applying the THP three-step model. Day 4: roleplaying module 1 sessions; handling difficult situations and adverse events; CHW supervision model; and THP implementation logistics.

### REP phases III and IV: implementation and maintenance and evaluation

While the focus of this article is on the steps leading up to the launch of THP, here we briefly describe the work conducted to prepare for booster trainings, supervision, and data collection on the implementation process to facilitate refinement of THP's next iteration at SES.

1. THP launch

The first perinatal woman began THP on 17 April 2017 after which nine additional women were enrolled as they entered the third trimester of pregnancy, the last beginning on 16 August 2017.

1. Booster trainings and supervision sessions

**Fig. 2.**
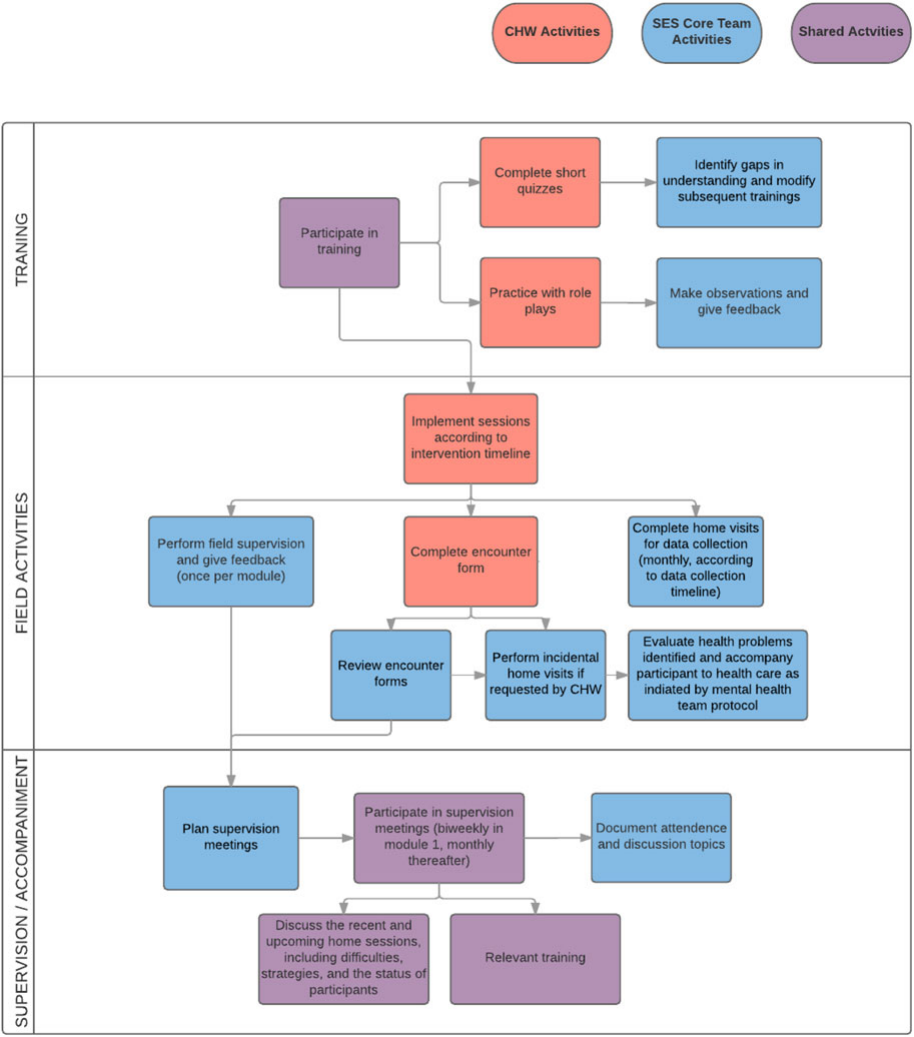
Planning, supervision, and accompaniment model planned for the implementation of the Thinking Healthy Programme in Lima, Peru.

We developed a CHW supervision plan with an accompanying data collection form (see Table 2) for CHWs to complete after every visit or attempted visit. Planned monthly CHW supervision meetings conducted by the mental health program coordinator will be conducted to discuss THP implementation issues, overcome problems/challenges encountered, and to offer supplemental training relevant to the THP modules that was not included in the initial training. Additionally, a social networking group (using the smart phone application Whatsapp^©^) for CHW and SES staff was established to maintain communication and to address concerns as they emerged. A field supervision plan consisting of regular observations by the SES mental health program coordinator of at least one session per module for all CHWs was also established, facilitating tailored feedback to the CHWs. A schematic overview of the supervision structure and intervention roles is shown in Fig. 2.

1. Planned data collection

Data collection began at enrollment prior to initiation of the first module for each mother enrolled into THP and will continue as indicated according to the data collection plan described in REP phase II, step 2, above.

## Results and discussion

The THP was implemented by a community-based organization in Peru with no prior experience in implementing CHW-delivered perinatal depression care. Pre-conditions for implementing THP were met given the need to address perinatal depression at the community level, the existing capacity of the SES mental health team, and the supportive resources available to adapt and carry out THP, an evidence-based, intervention delivered by non-specialists. In the pre-implementation phase, a systematic approach was followed, beginning with fully familiarizing the IT with the THP principles and content. Consultation with other THP implementers and experts in perinatal depression resolved uncertainties and informed training and implementation decisions. The pre-implementation prepared the IT to collect essential data that will inform future iterations of the intervention and help ensure that THP receives awareness and support within the health system. These steps were taken to facilitate expansion and endurance of THP beyond the original SES cohort of 10 women.

We learned how to train CHW in basic therapeutic skills important to this work (i.e. active listening, expressing empathy, not judging) and in the specific techniques of the cognitive–behavioral foundation of the intervention (identification and replacement of negative thoughts and behavioral activation). The didactic sessions were important in delivering the content and overall framework of THP to the CHWs, but direct feedback and peer evaluation of roleplays proved key to learning how to deliver the intervention. For example, in early roleplays, some CHW gave advice in the practice sessions, an approach that is required in some health interventions where clear, decisive direction is required. In THP, however, emphasis is placed on supporting the mother to generate alternative thoughts and practices that suit her lifestyle, with the CHW acting more as an empathetic coach than advice giver. Roleplays where two IT members demonstrated both styles – that of advice giver and that of empathetic coach – helped CHW contrast the two styles which they later practiced during the training.

Once the CHWs were prepared to deliver the intervention, they were embedded in a supportive accompaniment structure with planned supervision meetings, electronic communication, and encounter forms to be reviewed by the IT. Given that this was the first implementation of THP in the country, the IT will use the data collected to evaluate how THP works in the Peruvian context and will make recommendations for content or other changes once the first cohort of mothers complete the intervention. We also hope to include a qualitative evaluation of the mothers’ experiences and opinions of the intervention, the perspectives of participating mothers and their families, the CHW, and the health center staff. These data will be central in guiding future scale-up efforts (REP phase IV) and will provide critical information on the perceived value and drawbacks of THP by key stakeholders. Additionally, the clinical information collected (e.g. substance use; sleep disorders; intimate partner violence) may begin to inform potential modifications to THP and/or the need for new or adjunct community-based interventions. SES’s relationship and collaboration with the Ministry of Health and the local health center at each of the implementation phases described should pave the way for the scaling of THP beyond a single health center as the future lessons to be learned from the actual THP intervention are combined with our pre-implementation experiences thus far.

## Conclusion

This implementation of THP in Peru demonstrates the feasibility of introducing a non-specialist, packaged mental health intervention into a novel setting over a relatively short time frame: approximately 5 months from the decision to implement the intervention to the enrollment of the first perinatal woman. Implementing a mental health intervention into a novel setting delivered by non-specialists may appear daunting; however, by developing a plan to assess pre-conditions, develop pre-implementation plans for the intervention that draw on the experience and resources of other organizations, and beginning implementation at a small scale, THP can be delivered without previous experience for the benefit of perinatal women and their communities.
